# Pathologic complete response to KEYNOTE522 and HER2-directed therapy for synchronous TNBC and HER2+ breast cancer

**DOI:** 10.1038/s41698-024-00631-9

**Published:** 2024-07-28

**Authors:** Nicholas Mai, Jie-Fu Chen, Satshil Rana, Mark Robson, Sarat Chandarlapaty, Ezra Y. Rosen

**Affiliations:** 1 Department of Medicine, Memorial Sloan Kettering Cancer Center, New York, NY USA; 2 Department of Pathology, Memorial Sloan Kettering Cancer Center, New York, NY USA

**Keywords:** Breast cancer, Cancer genomics, Tumour heterogeneity

## Abstract

Simultaneous presentation of two separate primary breast cancers of differing histology at initial diagnosis is an uncommon phenomenon; it is even rarer to find these pathologically distinct populations within the same biopsy. Here we report the case of a patient diagnosed with clearly demarcated, pathologically heterogenous triple negative breast cancer (TNBC) and HER2+ breast cancer that was treated with a hybrid chemoimmunotherapy regimen combining elements of Keynote-522 and a standard HER2-directed neoadjuvant regimen, yielding apathologic complete response by the time of surgery with no notable adverse events. Molecular analysis of the histologically distinct tumor populations confirmed molecular evidence of differential HER2 expression but also suggested clonal relatedness of the two tumor populations based upon mutational profile, with phenotypic divergence potentially resulting from copy number alterations in *NF1*. Overall, this case highlights a rare histologic phenomenon that was successfully treated by combining both TNBC and HER2 directed neoadjuvant therapies.

## Introduction

Breast cancers are categorized by the presence or absence of cellular markers that are used to guide treatment: estrogen receptor (ER), progesterone receptor (PR), and human epidermal growth factor 2 (HER2). Breast cancer lacking expression of these three markers is known as triple negative breast cancer (TNBC). Standard of care neoadjuvant systemic therapy for triple negative breast cancer involves a combination of standard chemotherapy plus the PD-1 inhibitor pembrolizumab based upon the results of Keynote 522 (KN522)^1^. For early stage HER2 positive disease, neoadjuvant combinations of chemotherapy and HER2 directed therapy are the standard of care. However, there are no clear guidelines or recommendations when patients present with multiple primary breast cancer tumors of different subtypes. Further, while pembrolizumab and anti-HER2 agent combinations are used widely in other oncologic disease groups like GI malignancies, combining anti-PD1 and anti-HER2 therapies remains under active investigation in HER2+ breast cancer (including PANACEA^2^, KATE-2^3^, IMpassion050^4^, and CCTG IND.229^5^). To our knowledge, there are no trials nor reports of this combination for patients with synchronous HER2+ and triple negative breast cancer at presentation.

Here we report a patient with clearly demarcated, pathologically heterogenous HER2 + /TNBC breast cancer treated with a hybrid neoadjuvant regimen combining parts of a modified KN522 regimen with paclitaxel-carboplatin-trastuzumab-pertuzumab (TCHP), ultimately achieving a pathologic complete response at the time of surgery. Written consent to publish the information relevant to this case was obtained from the patient.

## Results

**Fig. 1:**
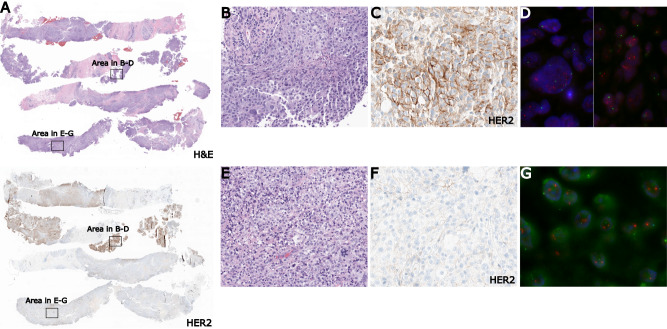
Pre-treatment biopsy showing subpopulations of Triple Negative and HER2+ Breast Cancer. **A** Biopsy identified high grade invasive breast carcinoma of no special type showing two distinct areas with differential expression of HER2. Both areas are negative for ER and PR. **B** H&E stain of representative HER2+ tumor population, with **C** 2 + HER2 expression by immunohistochemistry, and **D** low-level amplification by fluorescence in situ hybridization (FISH) with HER2/CEP17 ratio 2.4 and HER2 total copy 4.55/cell. **E** H&E stain of representative TNBC tumor population, with **F** 0+ to 1 + HER2 expression by immunohistochemistry, **G** which was negative by FISH with HER2/CEP17 ratio 1.32 and HER2 total copy 3.13/cell.

A 34-year-old female with at least cT3 breast cancer presented to our department for initial neoadjuvant chemotherapy evaluation. The patient had self-palpated a right breast mass, and a follow-up bilateral breast ultrasound in the same month showed a 4.3 cm lobulated hypoechoic mass, and US guided biopsy in the community noted breast carcinoma but tissue was inadequate to report HR or HER2 status. She subsequently underwent repeat biopsy which showed two distinct populations of tumor cells. Two of the four biopsy cores showed stronger HER2 expression by immunohistochemistry (IHC), and the other two cores showed a distinct tumor cell population that was considered triple negative breast cancer (Fig. 1A). Across the entire biopsy specimen, histologic examination showed poorly differentiated invasive breast carcinoma of no special type with solid growth pattern and high nuclear grade (III/III). Scattered foci of necrosis, pleomorphic nuclei, and atypical mitoses were noted. The overall tumor was associated with focal lymphoplasmacytic infiltrate, and estrogen receptor (ER) and progesterone receptor (PR) were negative (0% nuclear stain) by immunohistochemistry (IHC). The HER2-amplified tumor population showed confluent areas of immunoreactivity after HER2 staining (scored as 2 + ), accounting for approximately 50% of the tumor (Fig. 1B–D). Subsequent fluorescence in situ hybridization (FISH) analysis showed low-level amplification with a HER2/CEP17 ratio of 2.4 and HER2 total copy number at 4.55 per cell. In contrast, the TNBC tumor population exhibited 0+ to 1 + HER2 expression by IHC and was negative for amplification by FISH (HER2/CEP17 ratio 1.32 and HER2 total copy number 3.13/cell; Fig. 1E–G). Full staging workup, including bilateral breast MRI and PET CT showed no evidence of distant metastatic disease but did demonstrate a 6.9 cm enhancing right primary breast mass on MRI as well as an FDG avid right axillary lymph node. Within the 6.9 cm primary mass, MRI showed heterogenous enhancement but no specific radiographic features to suggest multiple discrete tumors. Her tumor was clinically staged as cT3N1M0, though follow-up US-guided biopsy of the FDG-avid axillary mass prior to further therapy was benign. Targeted genomic testing of both tumor and matched normal peripheral blood showed somatic mutations in *EZH2, MTOR, NF1, RAD21*,and *TP53* (Table 1), with no germline or somatic mutations in *BRCA1/2*. To optimize her neoadjuvant treatment given the histologic complexity of her tumor, we developed a novel hybrid regimen inspired by two standard of care neoadjuvant treatment regimens, KN522^1^ and TCHP, which are both commonly used in breast medical oncology.

**Table 1:** Somatic mutation profiling of separate tumor populations after macrodissection

Gene altered	RefSeq ID	cDNA change	Amino acid change	VAF in HER2-high area	VAF in TNBC area
EZH2	NM_004456	c.1706 C > T	p.A569V	0.33154	0.35636
MTOR	NM_004958	c.3649 G > C	p.V1217L	0.32414	0.20513
NF1	NM_000267	c.6862_6865delTCGC	p.S2288Lfs*9	0.66381	0.28792
RAD21	NM_006265	c.1644_1704+196del	p.X548_splice	0.62069	0.61069
TP53	NM_000546	c.679delT	p.S227Lfs*20	0.53281	0.44724

Both tumor populations shared the same SNVs/mutations but differed in VAF, overall indicating clonal origin despite notable phenotypic differences mentioned above. The clearest difference seen is in the VAF of *NF1* most likely due to copy number differences between the tumor populations.

**Fig. 2:**
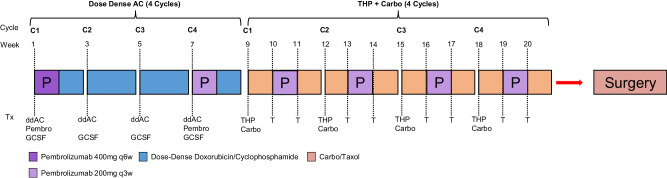
Schema for Modified KN522 + THP + Carbo Regimen. ddAC: Dose-dense doxorubicin (60 mg/m2) + cyclophosphamide (600 mg/m2) every 2 weeks; GCSF: granulocyte-colony stimulating factor, AKA peg-filgrastim; THP: weekly paclitaxel (80 mg/m^2^) and carboplatin (AUC 5) every 3 weeks, trastuzumab (8 mg/m2 loading followed by 6 mg/m2 thereafter) every 3 weeks, and pertuzumab (840 mg loading followed by 420 mg thereafter) given every three weeks.

The patient was initially treated with neoadjuvant dose-dense doxorubicin (60 mg/m^2^) + cyclophosphamide (600 mg/m^2^) (ddAC) every two weeks (with peg-filgrastim support) for four total cycles, given concurrently with pembrolizumab (400 mg) on cycle 1 day 1. Her pembrolizumab was transitioned to 200 mg every 3 weeks starting ddAC cycle 4 and continued at this dose until surgery. After ddAC, she started weekly paclitaxel (80 mg/m^2^) and carboplatin (AUC 5), trastuzumab (8 mg/m^2^ loading followed by 6 mg/m2 thereafter), and pertuzumab (840 mg loading followed by 420 mg thereafter) given every three weeks, with a total of four cycles of paclitaxel + carboplatin + HP + pembrolizumab (Fig. 2). She completed neoadjuvant chemoimmunotherapy without any dose-limiting adverse effects including cytopenias, though she did develop IO-mediated thyroiditis followed by hypothyroidism, for which endocrinology opted to observe due to her lack of associated symptoms. Interval mammography and breast MRI showed tumor response, with a decrease from 6.9 cm prior to treatment to 4.9 cm post-treatment. She ultimately underwent right mastectomy, and surgical pathology from surgery confirmed pathologic complete response in the primary site and no evidence of disease in sentinel lymph nodes (0/2). She has completed post-surgical radiation therapy, with plans to complete one year of adjuvant HP and at least 27 weeks of pembrolizumab (400 mg) given every 6 weeks.

## Discussion

We report the case of a patient with synchronous TNBC and HER2-amplified breast cancer successfully treated to pathologic complete response by time of mastectomy with a modified hybrid KN522 and TCHP neoadjuvant regimen. To our knowledge, this is the first report of a pathologic complete response for a patient with both triple negative and HER2+ breast cancer treated with the modified KN522 + TCHP regimen outlined above.

While the exact treatment combination that was used for this patient has not been studied in a randomized controlled trial for breast cancer, we do note that chemotherapy is routinely used in combination with PD1 blockade or in combination with anti-HER2 therapy separately. Further, anti-HER2 therapy coupled with PD-1 therapy is an established, safe treatment combination used for GI malignancies, and it is standard of care for first-line therapy of advanced HER2+ gastric cancer per the results of Keynote 811^6^. We extrapolated the safety and expected toxicities of this combination from our experience using it in gastric cancers, and the patient completed neoadjuvant treatment without any dose-limiting toxicities. We cannot exclude the possibility that her cancer, including the HER2+ components, may have responded to the chemotherapy in KN-522 alone even without HER2 targeting agents, but we believe that this treatment paradigm represents a superior treatment option for this patient as it is specifically tailored to the complex biology of her cancer.

**Fig. 3:**
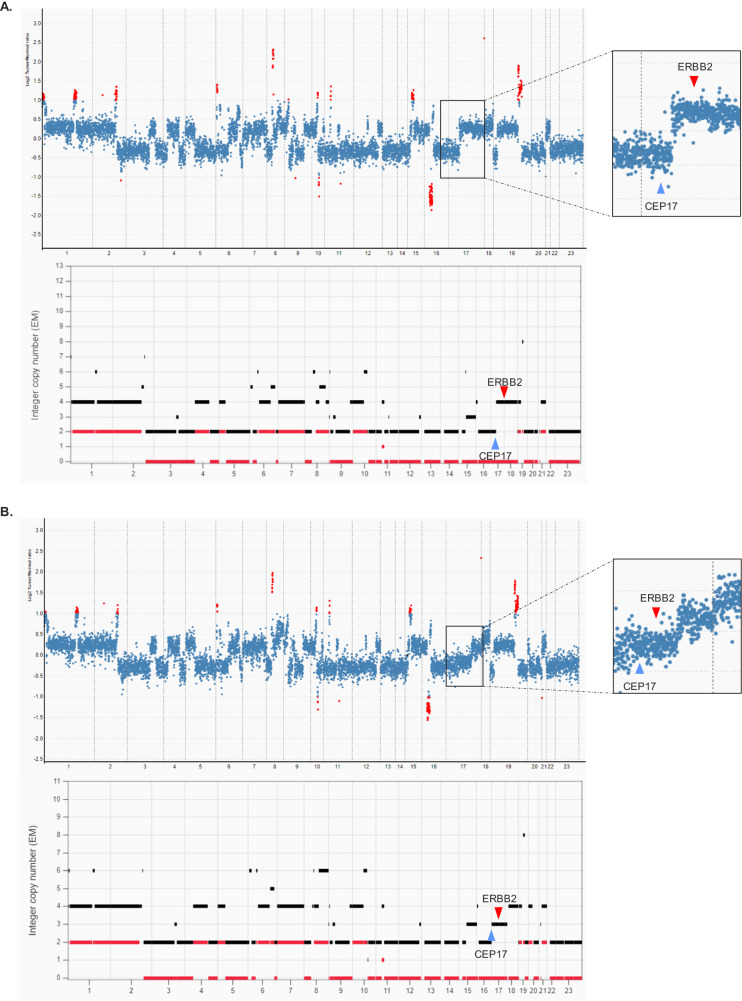
Copy number analysis of separate tumor populations after microdissection. Both areas shared almost identical copy number profile. The red dots indicated genes reaching significant level for amplification or deletion. Copy number profile and allele-specific copy number analysis by FACETS suggested widespread LOH and whole genome duplication. **A** Area with high HER2 expression showed copy number gain with chromosome arm 17q (including ERBB2) at 4 copies, whereas the area encompassing CEP17 remained at 2 copies, corresponding to a ERBB2/CEP17 ratio of 2. **B** Area with low HER2 expression showed 3 copies of chromosome 17 and a ERBB2/CEP17 ratio close to 1.

Original pathologic assessment of this patient’s tumor suggested the possibility of a collision tumor, which is a rare phenomenon where two distinct tumor areas with different expression patterns present in the same geographic location. To further characterize the molecular signatures of the two tumor populations, we macro-dissected the HER2 positive tumor population from the TNBC portion and submitted each component separately for somatic tumor mutation profiling via targeted next-generation sequencing (NGS) (MSK-IMPACT^7,8^) after she had already completed neoadjuvant therapy and surgery. Both areas shared the same single nucleotide variants/mutations (Table 1), including truncating mutations in *TP53* and *NF1*, a *RAD21* splice site variant that involves the 5’ canonical splice site of intron13, and heterozygous missense mutations in *EZH2* and *MTOR*. Both areas also shared a nearly identical copy number profile. Further analysis by allele-specific copy number analysis tool FACETS^9^ identified widespread loss of heterozygosity (LOH) and whole genome duplication, as indicated by integer copy number profiles (Fig. 3). Overall, the genomic sequencing findings indicate a common clonal origin for both tumor subpopulations, supported by the shared mutations and copy number profile. In addition to suggesting a common clonal origin, genomic sequencing identified significant differences in copy number of chromosome arm 17q between the HER2-amplified and TNBC components. In the HER2-amplified area, chromosome arm 17q (including *ERBB2*) showed copy number gain at 4 copies, whereas the area encompassing CEP17 remained at 2 copies (Fig. 3A). The entire chromosome 17 in the TNBC area showed slight copy number gain at 3 copies (Fig. 3B). The result of such copy number alteration resulted in a *ERBB2*/CEP17 ratio of 2 in the HER2-amplified area, and a *ERBB2*/CEP17 ratio close to 1 in the TNBC area, consistent with the initial data obtained by FISH.

The same focal copy number change in chromosome arm 17q also resulted in the discrepancy in variant allele frequency (VAF) of the *NF1* p.S2288Lfs*9 mutation, which is a truncating mutation at exon 46 that likely leads to disruption of NF1 protein. The VAF of this mutation in the HER2-amplified component was 66%, while the VAF in the TNBC component was 28.7%, both associated with LOH per FACETS analysis. In the HER2-amplified area, our analysis showed the focal copy number gain in chromosome arm 17q also involved exons 36 to 57 of *NF1* and elevated the VAF of the truncating mutation. In the TNBC component, *NF1* showed the same absolute copy number as *ERBB2* at 3 copies without focal copy number gain. While not definitive, our findings could suggest that her tumor possibly diverged phylogenetically to form these distinct subpopulations based on *ERBB2* and *NF1* copy number status. While there has been some suggestion that *NF1* loss of function mutations are associated with greater HER2 expression in breast cancers, it is unclear whether the differing *NF1* copy number in this tumor contributed to the difference in HER2 expression^10^.

LOH has been associated with homologous recombination deficiency (HRD) in some tumor types such as ovarian cancer or TNBC^11,12^, but multiple other mechanisms for widespread LOH have been demonstrated in other tumors^13–15^. We did not detect mutations in *BRCA1/2* genes or prominent mutation signatures associated with HRD^16^, and a HRD score was not available. While the possibility of underlying HRD cannot be entirely excluded, the etiology of widespread LOH in this tumor remains unclear given the available evidence.

Ultimately, while molecular analysis demonstrated that these were not technically two separate tumors, it did confirm our original pathologic and clinical assessment that the two tumor subtypes were distinct enough to be treated as two separate oncologic entities. Synthesizing all the clinical, pathological, and molecular data together, this is more likely a unique case of highly distinct intratumoral heterogeneity, which separately has been implicated in HER2-targeted therapy resistance^17^. These observations together show that standard treatments for TNBC and HER2+ breast cancer can be safely combined with excellent efficacy and tolerability in the neoadjuvant setting in patients with this unique histologic presentation.

## Methods

### Use of patient information

The patient provided written informed consent for her de-identified health information to be used in this report. She further consented to an Institutional Review Board approved protocol to perform tumor sequencing at Memorial Sloan Kettering. All physicians involved in this report complied with all relevant ethical regulations in patient interactions, in line with ethical norms and standards in the Declaration of Helsinki.

### Immunohistochemical stain and fluorescence in situ hybridization

Immunohistochemistry (IHC) and fluorescence in situ hybridization (FISH) was performed on 5-µm-thick sections from formalin-fixed, paraffin-embedded (FFPE) tissue utilizing antibodies for ER (6F11, Leica), PR (16, Leica), HER2/neu (4B5, Ventana), and enumeration probes for HER2 and CEP17 (HER2 IQFISH pharmDx, Agilent) per clinically validated protocol and interpreted per updated ASCO/CAP guidelines^18–20^. Distinct areas with differential HER2 expression were scored independently.

### Next generation sequencing

Targeted next generation sequencing on 505 cancer-related genes (MSK-IMPACT) was performed on FFPE tissue blocks of the tumor and matched peripheral blood as previously described^7,8^. The areas with different HER2 expression were manually macro-dissected on the unstained slides about HER IHC stained slide prior to DNA extraction and were processed independently. Somatic single-nucleotide variants (SNVs) were identified after germline variants detected in the paired normal sample were filtered out. The functional impact of detected mutations was classified using OncoKB^21^. Copy number analysis was performed with coverage-based method and with FACETS, an algorithm incorporating allele-specific copy number assessment and tumor purity estimation for detecting copy number alterations and loss of heterozygosity^9^.

### Reporting summary

Further information on research design is available in the Nature Research Reporting Summary linked to this article.

### Supplementary information

Reporting Summary

## Data Availability

Data from this study is available upon request to the corresponding author.
